# Emergence of polysaccharide membrane walls through macro-space partitioning via interfacial instability

**DOI:** 10.1038/s41598-017-05883-z

**Published:** 2017-07-21

**Authors:** Kosuke Okeyoshi, Maiko K. Okajima, Tatsuo Kaneko

**Affiliations:** Japan Advanced Institute of Science and Technology, 1-1 Asahidai, Nomi, Ishikawa 923-1292 Japan

## Abstract

Living organisms in drying environments build anisotropic structures and exhibit directionality through self-organization of biopolymers. However, the process of macro-scale assembly is still unknown. Here, we introduce a dissipative structure through a non-equilibrium process between hydration and deposition in the drying of a polysaccharide liquid crystalline solution. By controlling the geometries of the evaporation front in a limited space, multiple nuclei emerge to grow vertical membrane walls with macroscopic orientation. Notably, the membranes are formed through rational orientation of rod-like microassemblies along the dynamic three-phase contact line. Additionally, in the non-equilibrium state, a dissipative structure is ultimately immobilized as a macroscopically partitioned space by multiple vertical membranes. We foresee that such oriented membranes will be applicable to soft biomaterials with direction controllability, and the macroscopic space partitionings will aid in the understanding of the space recognition ability of natural products under drying environments.

## Introduction

By utilizing interfacial or mechanical instability, it is possible to control the geometrical structures of soft materials at the macro-scale^1–3^, *e.g*., fingering patterns of viscous liquids^4–7^ and buckling patterns of gels during swelling/deswelling processes^8–11^. These patterns are expected to be applied in dynamic materials having smart functions such as in cell manipulation, capture/release, and mass transport in biomedical fields^12–15^. For this, evaporative self-assembly on air-liquid/solid-liquid interfaces has been widely used for the preparation of spatially ordered macro-structures such as colloidal crystals^16–22^. The drying process plays crucial roles in the integration of the structural unit for macroscopic patterns such as stripe and ring shapes^23–30^. In nature, to adapt to drying environments, many types of regularly-patterned wet structures, such as plant cell walls and skin tissues, have emerged. In fact, living tissues have macroscopically anisotropic structures, such as vascular bundles for the directional control of water, and they have spatially partitioned structures in macro-scales, such as multi-cellular assemblies and tree branching. However, the effect of drying environments on these dissipative structures either *in vivo* or *in vitro* has not yet been unveiled.

Here, we use a polysaccharide, *sacran*, extracted from one of the cyanobacteria, *Aphanothece sacrum*, which has an extremely high molecular weight (*M*_w_ > 2 × 10^7^ g·mol^−1^) and is ten times thicker than typical thickeners such as hyaluronic acid (*M*_w_ ≈ 10^5^ g·mol^−1^) (Fig. S1) ^31–35^. This polymer was reported to be a functional and unique biomaterial, with super-moisturization, lyotropic liquid crystallinity, and anisotropic hydrogel swelling properties. Recently, we discovered that the aqueous liquid crystalline (LC) solution of *sacran* in drying conditions exhibited polymeric assembles of huge rod-like microdomains with a diameter of ~1 µm and a length of greater than 20 µm as the structural unit of the LC^36^. These dimensions are much greater than those of cellulose nanocrystals, with a typical outer diameter of 5 nm and length less than 1 µm^37, 38^. The *sacran* microdomains were fused and oriented parallel to the air-LC interface to form a single milliscale macrodomain in drying conditions. The orientation starts from the interface to form a densely accumulated structure similar to that of the skin layer of a deswelling gel with shear stress in mechanical instability^6, 39^.

In this study, we report a non-equilibrium process of the polysaccharide LC solution through deposition and hydration during drying in a limited space. Our strategy for the control of the non-equilibrium state is based on the idea that drying induces the integration of the LC structural unit to form a single macrodomain at the milliscale at the air-LC interface^36^. The condensed macrodomain at the interface would suppress evaporation, and the situation can potentially induce a thermodynamic dissipative structure. To initialize the evaporation front, *i.e*., the geometries of the fluid phase for drying, the LC solutions are evaporated from top-side-open cells with a narrow gap. We derived a deposition law for forming a hierarchical megalo-structure over time in three-dimensional spaces.

## Results and Discussion

### Emergence of vertical membrane via interfacial instability

**Figure 1:**
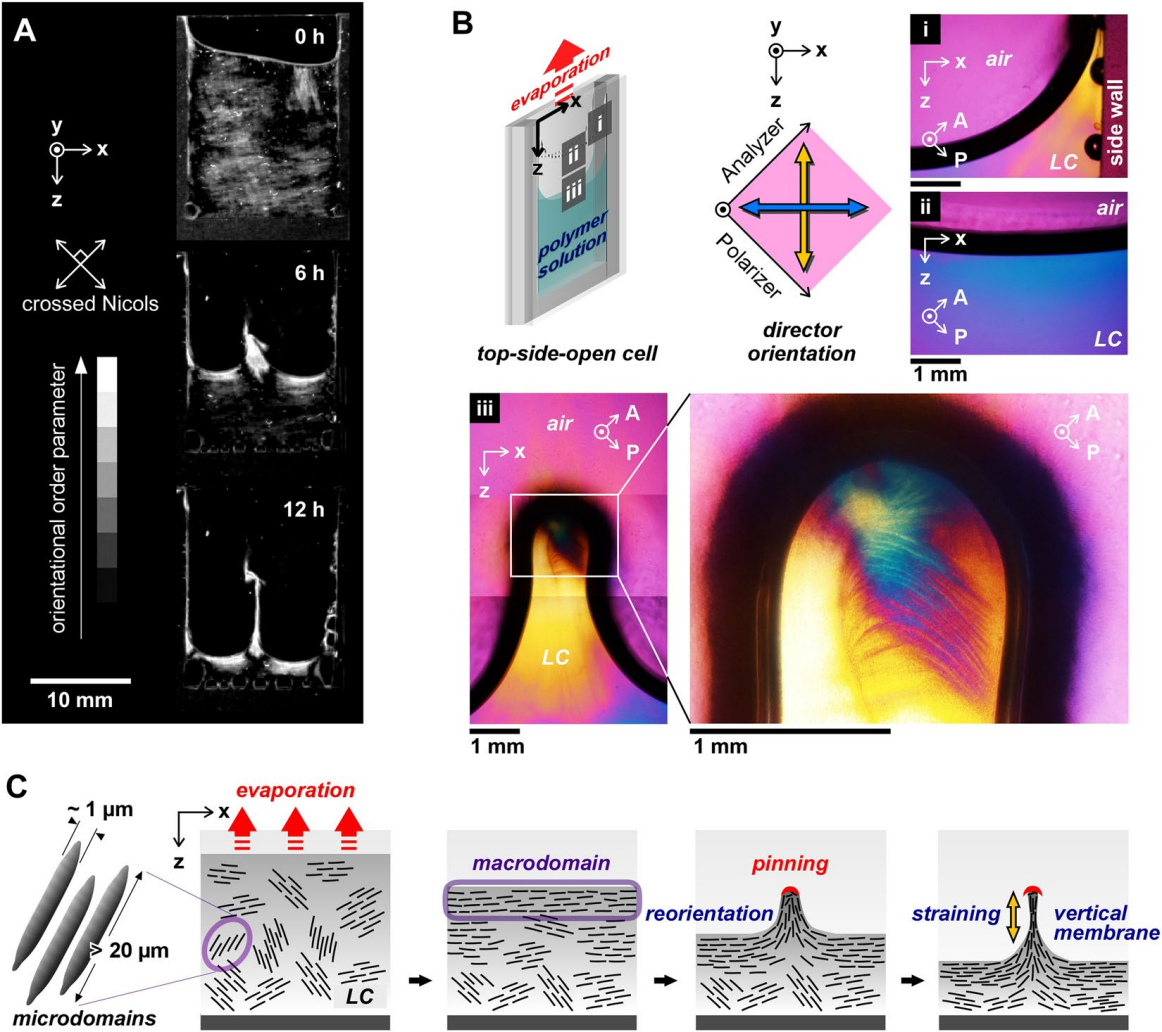
Interfacial instability of the polysaccharide liquid crystalline (LC) solution during drying in limited space. (**A**) Side views of a *sacran* solution during drying process under cross-polarized light. (**B**) Polarized microscopic images of the solution near the air-LC interface after 3 h (area i and ii) and 6 h of drying (area iii). The images were observed through a retardation plate, λ = 530 nm with crossed Nicols in the illustrated directions. (**C**) Schematic illustration for formation of vertical membranes by drying of a LC polymer solution in limited space with a narrow gap. Rod-like microdomains orient from the air-LC interface parallel to the contact line in the drying process. By drying in limited space, the non-equilibrium state between deposition and hydration near the interface causes pinning and vertical membrane formation between the narrow gap. The *Z*-direction is parallel to the direction of gravity. The gap between glass slides is *∆y*_0_ = 1 mm. Initial concentration of *sacran*: 0.5 wt%. Drying atmosphere: 60 °C under air pressure.

The viscous solution was poured into a top-side-open cell composed of two glass slides and a silicon spacer with an *X*-width of 15 mm and a *Y*-thickness (*∆y*_0_) of 1 mm at ~25 °C. The initial concentration was 0.5 wt%, which was higher than the critical concentration of LC formation (*~*0.2 wt%)^33^. The solution has the extremely-high viscosity at the initial state (dynamic moduli of the 0.5 wt% solution at 1 Hz frequency: storage moduli, *G*′ ≈ 4 Pa; loss moduli, *G*″ ≈ 2 Pa)^32, 33^. The sample was placed at 60 °C where the LC state was stably kept under atmospheric pressure in an oven with an air circulator. The evaporation rate was optimized by controlling the temperature to avoid bubbles generation and to make an affective air-LC interface^36^. The drying process was monitored under cross-polarized light from the side (Fig. S2). As shown in Fig. 1A, in the initial state at room temperature, several milliscale macrodomains were observed, showing liquid crystallinity. Just after drying, the transmitted light intensity was significantly increased around the interface, suggesting that the rod-like microdomains with lengths of several tens of a micrometer were integrated from the air-LC interface in parallel by capillary force^36^. This integration to form a single macrodomain on the air-LC interface was also confirmed on the whole region, including the side wall, using a polarized microscope with a retardation plate (λ = 530 nm) (Fig. 1B, area i–ii).

In addition, light transmitting was slightly detected on the glass surface just above the three-phase contact line owing to the oriented polymer adsorption onto the glass substrates (Movie S1). This result suggested that drying induced not only self-integration from an air-LC interface but also adsorption on a solid surface with an oriented state. Here, this adsorption became the nucleus for the deposition to show pinning and a vertical membrane wall in the *Z*-direction during drying. In this report, the cell was composed of non-modified glass slides, and its surface chemistry effect is currently under investigation. To clarify the direction of the microdomains in the pinning process, the interfacial region near the glass surface was observed using a polarized microscope (Fig. 1B, area iii). The yellow regions of the pinning on the liquid phase were significantly oriented in the *Z*-direction, while the top of the pinning showing blue indicates that the phase included an oriented structure in the *X*-direction. This means that the integrated structure dynamically changed the director orientation from the *X*-direction to the *Z*-direction with the formation of pinning (Fig. 1C). Such formation of a vertical membrane could be seen in the *Y*-direction of the cell, *∆y*_0_ < 1 mm, and there appeared to be a critical thickness for membrane formation. As the *∆y*_0_ decreased to 1 mm, the area of the air-LC interface decreased, inhibiting evaporation with deposition on the walls and inducing pinning. The same drying experiment was conducted with a *xanthan gum* solution, which has a lower *M*_w_ = 4.7 × 10^6^ g·mol^−1^, showing the LC state with an initial concentration of 0.5 wt% (Fig. S3). The deposited nuclei of the *xanthan gum* emerged in a cell with *∆y*_0_ of 1 mm, but the vertical membrane broke loosely during the formation. The difference between these two polysaccharide solutions was apparently due to the mobility of the structural unit of the LC state in the drying process. The *sacran* solution has lower mobility while the *xanthan gum* solution has higher mobility, which is an important consideration for the vertical membrane formation process^36^. Because *xanthan gum* solution showed a vertical membrane after tuning the *∆y*_0_ to ~0.5 mm, the formation of vertical membranes may be a universal phenomenon exhibited by polysaccharides (see Fig. S4).

### Unidirectional orientation in vertical membrane

**Figure 2:**
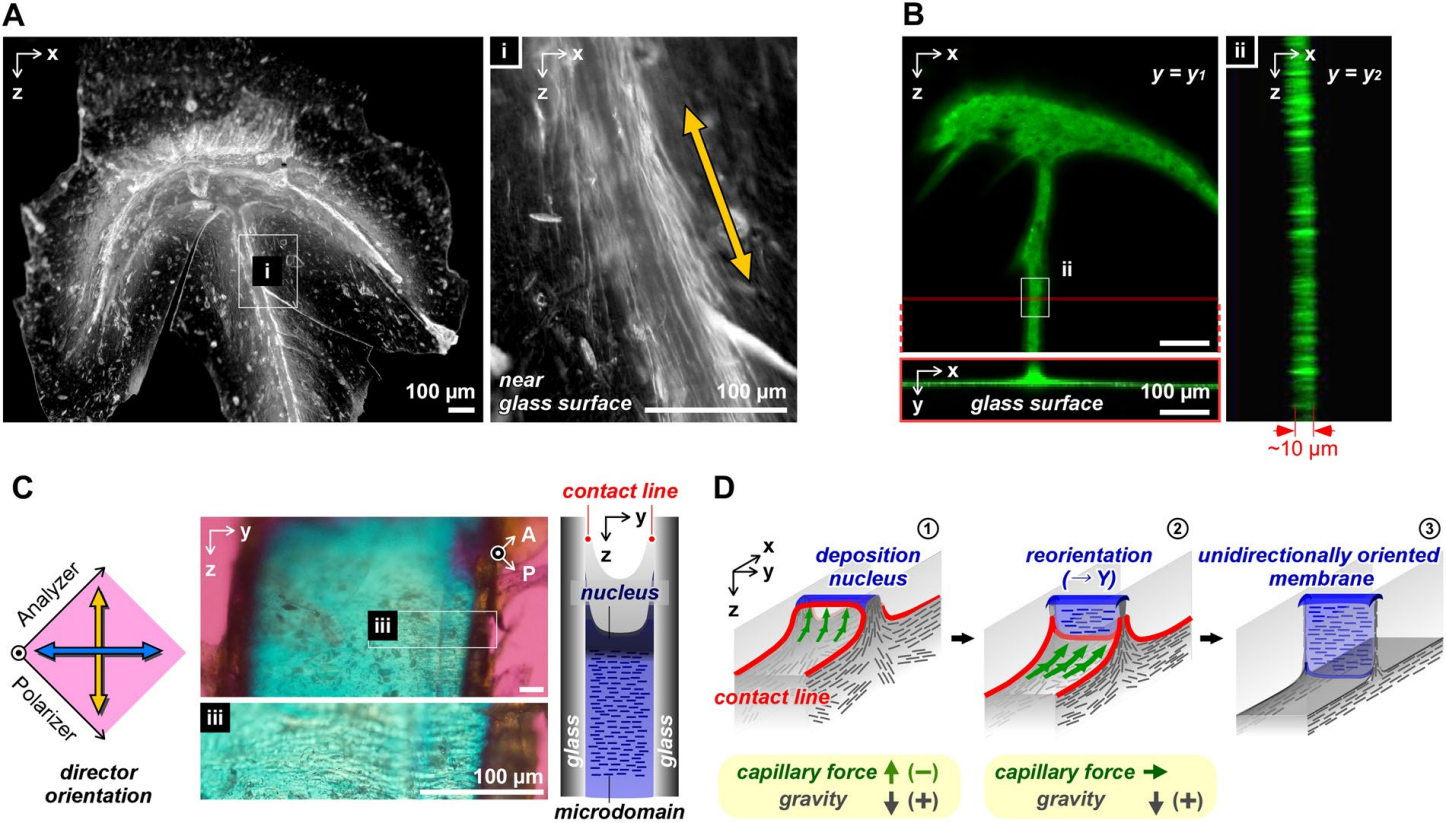
Microscopic images of a nucleus and the vertical membrane formed between glass slides. (**A**) Optical microscopic images of a dried membrane between glass slides observed from the *XZ*-plane. All scale bars = 100 µm. (**B**) Confocal microscopic images of a dried membrane prepared from a FITC-conjugated *sacran* solution. All scale bars = 100 µm. (**C**) Polarized microscopic images of the membrane in the *YZ*-plane observed through a retardation plate, λ = 530 nm and schematic illustration of the vertical membrane formed between the glass slides. All scale bars = 100 µm. (**D**) Schematic illustration of a unidirectionally oriented membrane formation utilizing the air-liquid-solid phase contact line during the drying process in limited space. 1) Just after generation of nucleus, which links the glass slides, the capillary force on the nucleus is in the direction opposite that of gravity. 2) After formation of the nuclei-cap, the effect of the capillary force in the *Z*-direction is relieved. 3) The microdomain integrates with the contact line to form a unidirectionally oriented membrane.

After pinning, the deposited polymer interlinked the glass template surfaces and a thin membrane grew in the *YZ*-plane. Figure 2A,B shows the microscopic images of a dried vertical membrane around the nuclei observed by optical microscopy and confocal microscopy using fluorescein-4-isothiocyanate isomer (FITC)-conjugated *sacran*. The nuclei formed an arrow shape and the membrane in the *YX*-plane near the glass surface showed fiber structures parallel to the *Z*-direction (Fig. 2A, area i). These results support the deposition mechanism along the three-phase contact line, as shown in Fig. 1D. By confocal microscopy, the thickness of the vertical membrane in the *YZ*-plane was confirmed to be ~10 µm (Fig. 2B, area ii and Fig. S5). To reveal the orientation, the membrane was observed by polarized microscopy in the *YZ*-plane (Fig. 2C). The membrane showed a significant blue color, suggesting that the orientation was along the *Y*-direction, and creases were observed in the *Y*-direction (Fig. 2C, area iii). Considering that the length of the rigid microdomain was more than 20 µm and the membrane thickness was ~10 µm, the microdomain may have been unidirectionally oriented along the *Y*-direction.

On the basis of these results, the formation of an oriented membrane between plane surfaces is schematically illustrated in Fig. 2D. After the deposition to form nuclei, the contact line on the nuclei included a stronger vector in the *Z*-direction. Herein, strains with opposite directions exist, *i.e*., the capillary force on the nuclei is in the *Z*(−)-direction (upward) and the gravity is in the *Z*(+)-direction (downward). This situation induced the straining of the macrodomain in the *Z*-direction, which resulted in a pinned state. After formation of the nuclei-cap, the effect of the capillary force in the *Z-*direction is relieved (Fig. S6). In the condition where the capillary force included a weaker vector in the *Z*-direction, the microdomains easily reoriented in parallel to the line without gravity effects. As a result, the vertical membrane was formed with an oriented structure in the *Y*-direction along the contact line.

**Figure 3:**
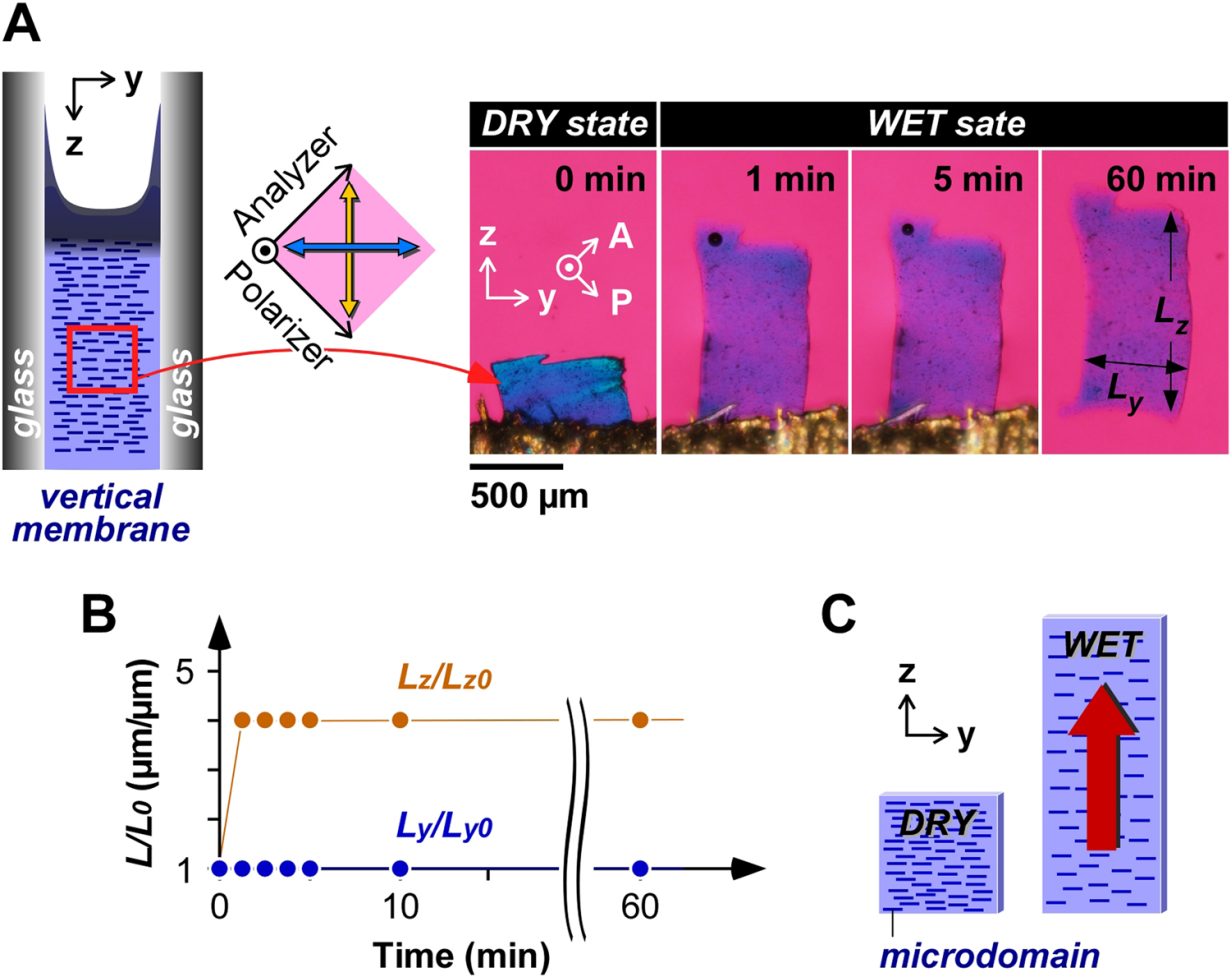
Unidirectional swelling of the crosslinked membrane. (**A**) Polarized microscopic images in swelling process of crosslinked membrane. (**B**) Swelling kinetics of the membrane in the *Y*-direction and the *Z*-direction. (**C**). Schematic illustration of the unidirectional swelling of the membrane. *L*_0_ indicates the initial length of the membrane in the dried state. *L*_y_ is the length of the membrane in the *Y*-direction; *L*_z_ is the length of the membrane in the *Z*-direction.

Furthermore, crosslinking points can be introduced into the dried polysaccharide membrane by annealing at more than 80 °C^34–36^. Once water is removed by heating, the polysaccharides can form multiple hydrogen bonds, which leads to the physical crosslinking of the chains. When a few ester bonds of uronic carboxylic acid with hydroxyls are formed, these covalent bonds should work as chemical cross-linking points^34^. It is also possible to peal off the dried vertical membrane from the cell while retaining the integrity (Fig. S7). By cutting the dried membrane to obtain the middle parts, the swelling property was investigated. Figure 3A shows the swelling process from a dry state to a wet state of a crosslinked membrane observed using a polarized light microscope. The crosslinked membrane quickly swelled by ~4-fold only in the *Z*-direction in less than 1 min, but not in the *Y*-direction, while maintaining orientation (Fig. 3B). The uniaxial swelling parallel to the planar direction indicates expansion of the interval on the short axis of the rod-like microdomains (Fig. 3C). This swelling behavior of a quasi-1D hydrogel is geometrically different from that of a hydrogel prepared from a dried film on the bottom, which swells vertical to the plane (Fig. S8)^34, 35^. The swelling kinetics of the film with planar orientation on the bottom should be affected by the size especially in the *XY*-plane. In contrast, that of the vertically-formed membrane with linear orientation in this report should not be affected by the size in any dimension. Such a hydrogel would be useful as a biocompatible soft actuator with a quick response.

### Macro-space partitioning by membrane walls

**Figure 4:**
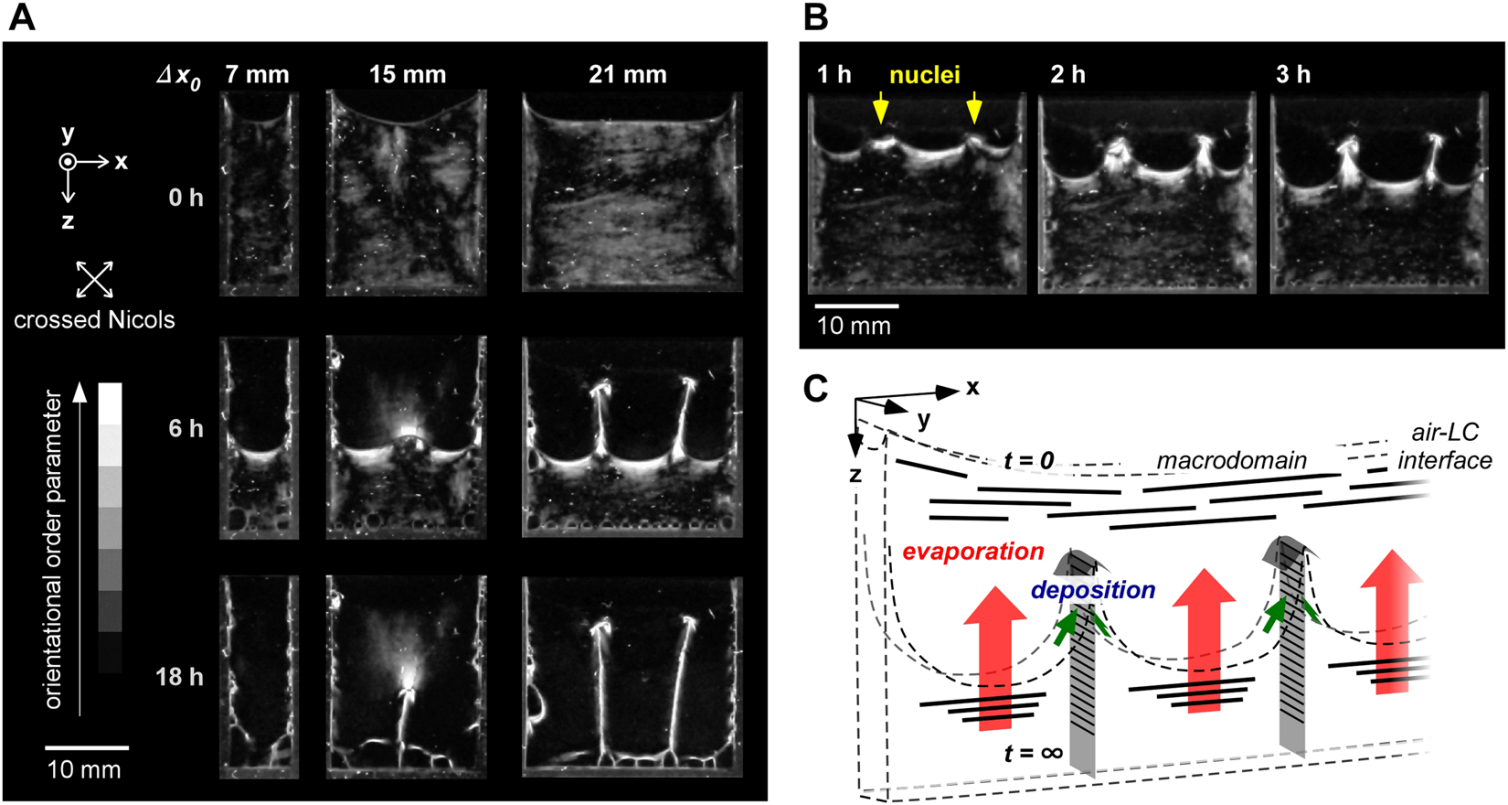
Multiple nuclei deposition to form vertical membrane walls and macro-space partitioning. (**A**) Time courses of vertical membrane formation during drying from top-side-open cells with *X*-width, *∆x*_0_ = 7, 15, and 21 mm. The images were acquired under cross-polarized light. (**B**) Initial time course of the nucleation from the cell with *X*-width, *∆x*_0_ = 21 mm. The *Y*-thickness is *∆y*_0_ = 1 mm. Initial concentration of *sacran*: 0.5 wt%. Drying atmosphere: 60 °C under air pressure. **C**. Mechanism of multiple vertical membrane formation through non-equilibrium state between deposition and hydration during drying in limited space.

To illuminate the non-equilibrium state between the deposition and the hydration on the interface, the *Y*-thickness, *∆y*_0_, of the top-side-open cell was fixed at 1 mm and the *X*-width, *∆x*_0_, was adjusted. As shown in Fig. 4A and Movie S2, although no expression of a vertical membrane wall was shown from a cell with an *X*-width of 7 mm, one vertical membrane was expressed from that with an *X*-width of 15 mm, and two from that with an *X*-width of 21 mm. These phenomena clearly demonstrate that during drying, the solution experiences interfacial instability, which induces multiple nucleation for the deposition. The formation of multiple nuclei apparently follows the same mechanism as single nucleation, which forms an arrow-shaped nuclei-cap and vertical membrane walls (Fig. 4B).

The formation of multiple nuclei is supposed to be related to a balance between the evaporation rate and deposition rate (Fig. 4C). Considering that the evaporation rate was almost the same in different *X*-widths (see Fig. 4A), the deposition rate should be independent on the *X*-width. However, a dense layer on the interface suppressed the evaporation even when the same amount of thermal energy was continuously supplied. With an increase in the *X*-width, the polymer deposition rate on the side walls was not sufficiently high, causing additional deposition at specific positions. This deposition relieved the suppression of evaporation without further thickening of the dense layer at the interface. This non-equilibrium state between hydration and deposition is supposed to induce the multiple deposition points macroscopically according to the following equation (Fig. S9):

1 $${{\rm{C}}}_{{\rm{i}},{\rm{t}}}=\frac{{\rm{Weight}}\,{\rm{of}}\,{\rm{dissolved}}\,{\rm{polymer}}\,{\rm{in}}\,{\rm{a}}\,{\rm{partitioned}}\,{\rm{space}}}{{\rm{Volume}}}\approx \frac{{{\rm{W}}}_{{\rm{i}}}-{{\rm{\alpha }}}_{{\rm{i}},{\rm{t}}}}{{{\rm{\Delta }}x}_{{\rm{i}}}{{\rm{\Delta }}y}_{0}{{\rm{\Delta }}z}_{{\rm{i}},{\rm{t}}}}$$

(*C*_*i,t*_: polymer concentration in a partitioned space at time *t*, *W*_*i*_: total weight of dissolved and deposited polymer in a partitioned space, *α*_*i,t*_: deposition weight of polymer on inside walls and vertical membranes, *∆x*_*i*_: width of a partitioned space between membranes, *∆y*_0_: the *Y*-thickness of the liquid phase, *∆z*_*i,t*_: height of liquid phase at time *t*). Although the *C*_*t*_ at the non-equilibrium state was not spatially homogeneous, depending on the distance from the interface, the saturated concentration, *C*_*s*_ was dominant near the interface. When the concentration near the interface became saturated (*C*_*i, t*_ (*z* = *∆z*_*0*_ − *∆z*_*i,t*_) > *C*_*s*_), deposition occurred on the glass surfaces and at the gap between the glasses to grow vertical membranes according to the equation (1), *α*_*i,t*_ ≈ *W*_*i*_ − *C*_*i,t*_ *∆x*_*i*_ *∆y*_0_ *∆z*_*i,t*_. The macrodomains’ position in the lower liquid phase did not vary much from the initial LC state (see Fig. 4B), meaning that the concentration change in the lower phase by thermal convective heat was insignificant. Thus, macro-space partitioning was induced on the spatially limited air-LC interface.

**Figure 5:**
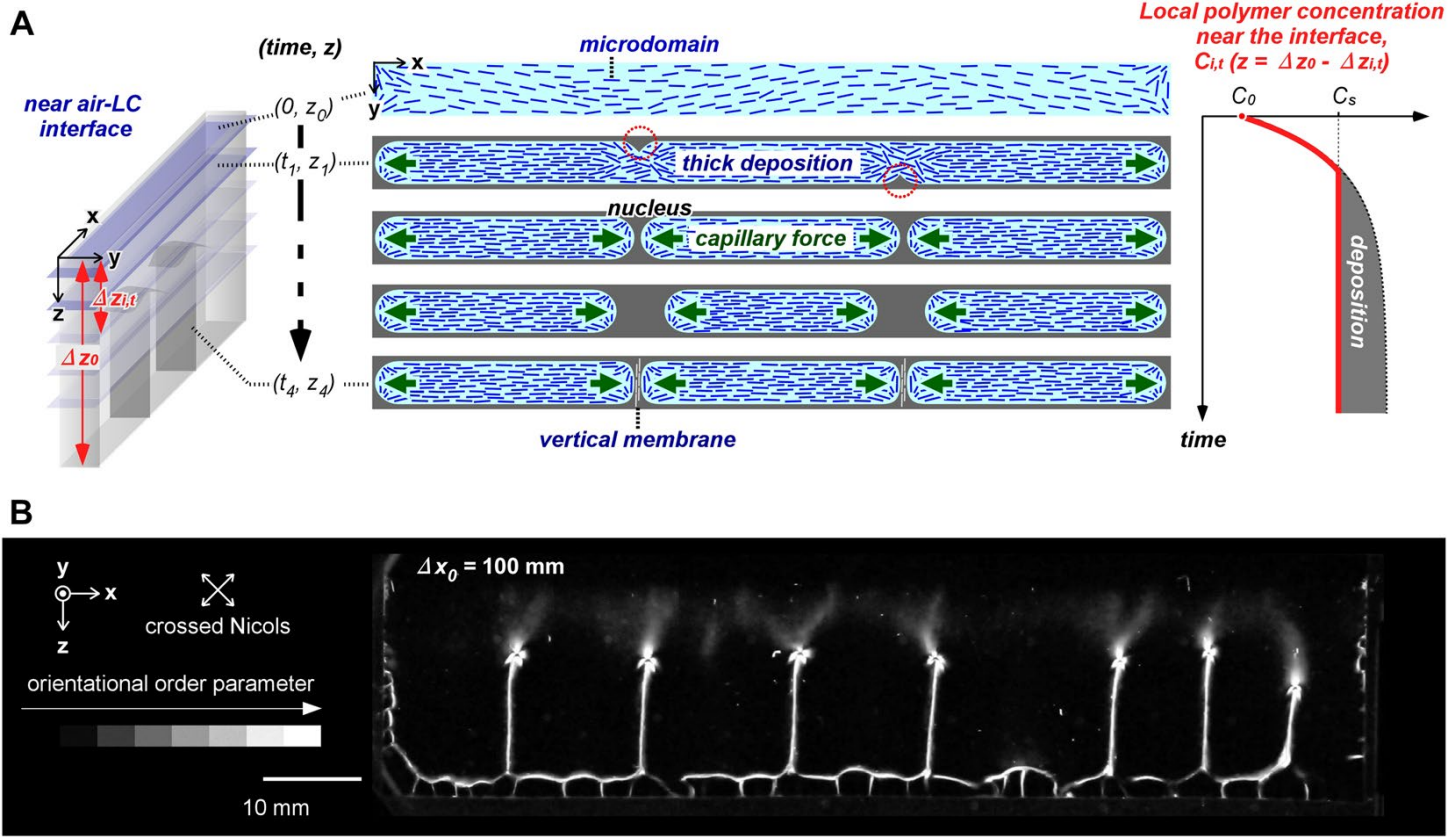
Macro-space partitioning via membrane walls in top-side-open cell. (**A**) Schematic illustrations of cross-sectional views in the *XY*-plane near the air-LC interface, and the change in polymer concentration in time course. When the concentration is saturated at the interface, the polymer is deposited at several specific points as thick nuclei on the walls. These points grow into a link between the slides, and the capillary force enhances the formation of vertical membranes. *C*_0_: initial concentration; *C*_S_: saturated concentration; *C*_*i,t*_: local polymer concentration near the interface in a partitioned space *i* at time *t*. (**B**) Macro-space partitioning by multiple vertical membranes dried in limited space with *X*-width, *∆x*_0_ = 100 mm. The *Y*-thickness is *∆y*_0_ = 1 mm. Initial concentration of *sacran*: 0.5 wt%. Drying atmosphere: 60 °C under air pressure.

Figure 5A shows the schematic illustration of cross-sectional views in the *XY*-plane near the air-LC interface, especially focusing on the nucleation. During the drying, microdomains deposits along the contact lines in the cell at the beginning. 1) Thick deposition at an arbitrary position enhanced subsequent deposition, resulting in formation of multiple nuclei and interlinking between the glass slides. 2) Around the nucleus, evaporation induced further deposition by capillary force to form a nuclei-cap. 3) The rod-like microdomains were integrated along the contact line of the nucleus via capillary force to form the vertical membrane. 4) As a result, multiple membrane walls were formed. Furthermore, we observed more than two membrane walls by increasing the *X*-width, as shown in Fig. 5B. This result determinably supports the interfacial instability that the non-equilibrium state between deposition and hydration on the air-LC interface induced multiple nucleation and membrane formation. As one of the non-equilibrium states^1, 2^, the dissipative structure was ultimately immobilized as macroscopically partitioned spaces by the multiple vertical membranes.

## Conclusion

We demonstrated multiple nucleation of a mega-molecule, the polysaccharide *sacran*, by drying an aqueous LC solution in limited space, to grow vertical membrane walls with unidirectional orientation. The rod-shape LC microdomain with a diameter of ~1 µm and length of greater than 20 µm were organized into a membrane with the dimensions of 1 mm × more than 15 mm, showing macro-space partitioning on the centimeter-scale. The dissipative structure through a non-equilibrium state between deposition and hydration was immobilized as a macro-structure having an order of the LC microdomain. In fact, the unidirectionally oriented membranes were successfully prepared at the millimeter-scale through reorientation of the microdomain along the contact line (orientation direction: *X* → *Z* → *Y*). Consequently, the thermally crosslinked membrane exhibited uniaxial swelling parallel to the planar direction. This type of quasi-1D hydrogel will be applicable as wet materials with directional controllability such as anisotropic semi-permeable membranes, artificial cell walls, and cell scaffolds involved in molecular signaling pathways. Furthermore, we envision that the macro-space partitioning in drying will aid in the understanding of space recognition ability in natural products under interfacial instabilities.

## Methods

### Materials

*Sacran* was extracted from *Aphanothece sacrum* according to previous work^31^. *Xanthan gum* extracted from *Xanthomonas campestris* was purchased from Taiyo Kagaku Co., Japan. FITC-I was purchased from Dojindo Molecular Technologies Inc., Japan. Top-side-open cells were prepared with two glass slides and a 1-mm-thick silicon spacer.

### Preparation of polymer solutions

After dissolving *sacran* in pure water at ~80 °C, it was cooled at room temperature to obtain aqueous solutions with a concentration of 0.5 wt%. This concentration was higher than that of liquid crystal phase transition (>*~*0.2 wt%)^33^. The *sacran* solution was centrifuged to remove insoluble impurities (Beckman equipped with JA-20 rotor, 2 × 10^4^ rpm, 4 °C, 1 h: three times).

### Drying experiments and observations under cross-polarized light

The sample aqueous solutions were poured into a top-side-open cell composed of non-modified glass slides and a silicon spacer at room temperature, and they were placed in an oven (60 °C) under atmospheric pressure with an air circulator^40^. The glass slides were cleaned by washing with acetone and spraying air using an air gun. To verify the degree of orientation in time course, samples were photographed through linear cross-polarizers (Fig. S2). The transmitted light intensity was analyzed by ImageJ to evaluate the degree of orientation spatially. Polarized microscopic observations were made using a microscope (BX51, Olympus) equipped with a CCD camera (DP80, Olympus). A first-order retardation plate with λ = 530 nm was put onto the light path. Confocal microscopic observations were made using a microscope (FV1000D-IX81, Olympus).

## Electronic supplementary material

Supplementary Information

Movie S1

Movie S2
