# Supplementary material for: Emergence of polysaccharide membrane walls through macro-space partitioning via interfacial instability
Source: Sci Rep. 2017 Jul 21;7:5615. doi: 10.1038/s41598-017-05883-z (PMC5522447; doi:10.1038/s41598-017-05883-z)
Supplement: Supplementary file 1 — Supplementary Information [file 41598_2017_5883_MOESM1_ESM.pdf]

**Supplementary Information**

# Emergence of polysaccharide membrane walls and macro-space partitioning via interfacial instability

*Kosuke Okeyoshi*, Maiko K. Okajima, and Tatsuo Kaneko**

Japan Advanced Institute of Science and Technology,

1-1 Asahidai, Nomi, Ishikawa 923-1292, Japan

E-mail: okeyoshi@jaist.ac.jp; kaneko@jaist.ac.jp

**Supporting Movie S1.** (see **Figure 1A**)
Drying process of the 0.5 wt% *sacran* solution at 60 ˚C from a top-side-open cell with $X$-width, $\Delta x_0$ = 15 mm. Other dimensions of the cell: $Y$-thickness, $\Delta y_0$ = 1 mm and $Z$-height, $\Delta z_0$ = ~20 mm.

**Supporting Movie S2.** (see **Figure 4A**)
Drying process of the 0.5 wt% *sacran* solution at 60 ˚C from top-side-open cells with $X$-width, $\Delta x_0$ = 7 mm, 15 mm, and 21 mm. Other dimensions of the cell: $Y$-thickness, $\Delta y_0$ = 1 mm and $Z$-height, $\Delta z_0$ = ~20 mm.

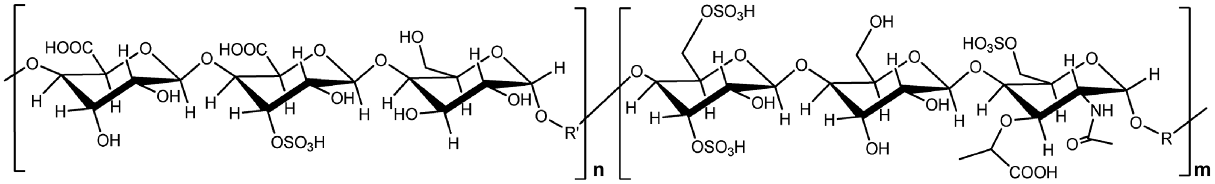

***Figure S1*. Chemical structure of polysaccharide, *sacran*.**

Elemental analyses and chromatographic and spectroscopic studies of *sacran* revealed the following sugar residues: Glc, Gal, Man, Fuc, Rha, Xyl, Rib, methylated hexose, uronic acids, and trace muramic acid. The carboxylate composition was 11 mol%, and substitution of sulfate groups was favored when the sulfate composition was 22 mol% with sugar residues.[1]

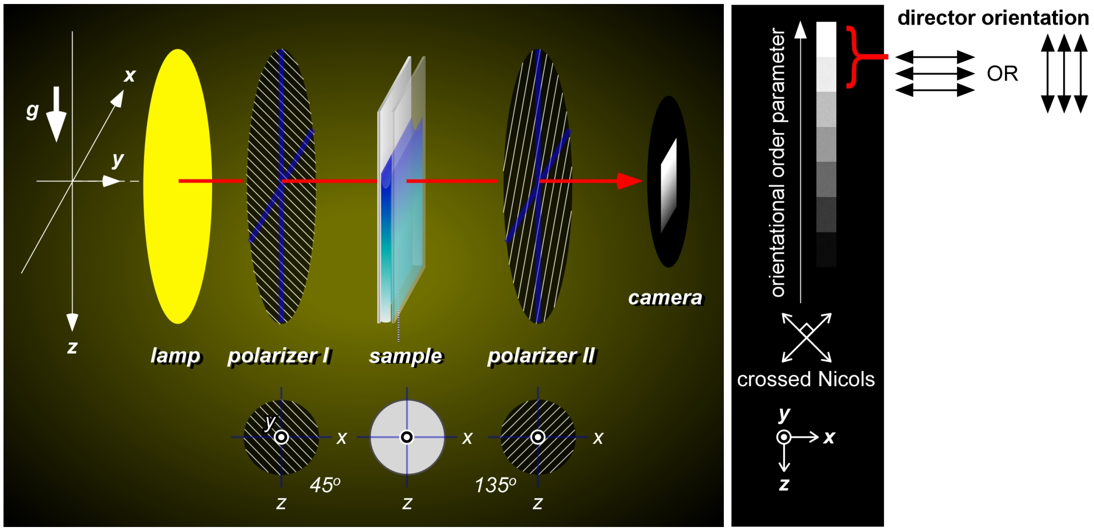


***Figure S2*. Schematic illustration of the experimental setup used for observations under cross-polarized light.** The polarizers were normally adjusted to 45° and 135°.

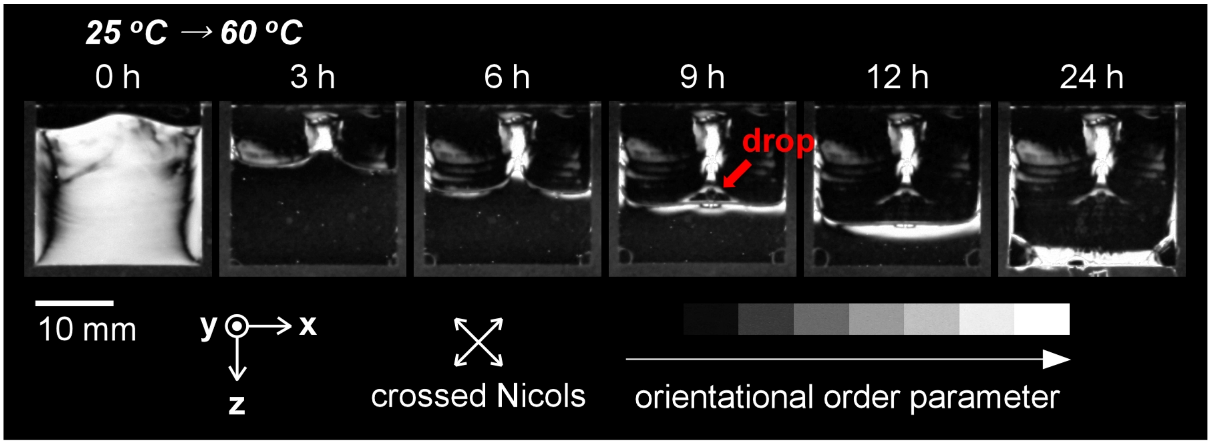


***Figure S3*. Drying process of a *xanthan gum* solution from a top-side-open cell with *X*-width. The images were acquired under cross-polarized light.** Dimensions of the cell: ($\Delta x_0$, $\Delta y_0$, $\Delta z_0$) = (21 mm, 1.0 mm, ~20 mm). Initial concentration: 0.5 wt%. Drying atmosphere: 60˚C under air pressure.

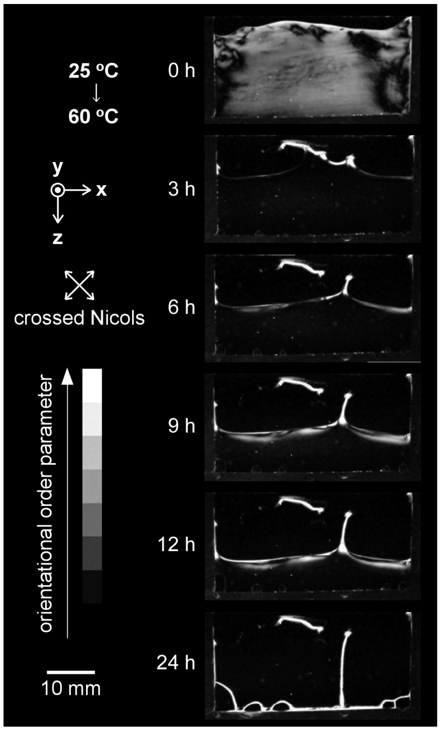


***Figure S4*. Drying process of a *xanthan gum* solution from a top-side-open cell. The images were acquired under cross-polarized light.** Dimensions of the cell: ($\Delta x_0$, $\Delta y_0$, $\Delta z_0$) = (42 mm, 0.5 mm, ~20 mm). Initial concentration: 0.5 wt%. Drying atmosphere: 60˚C under air pressure.

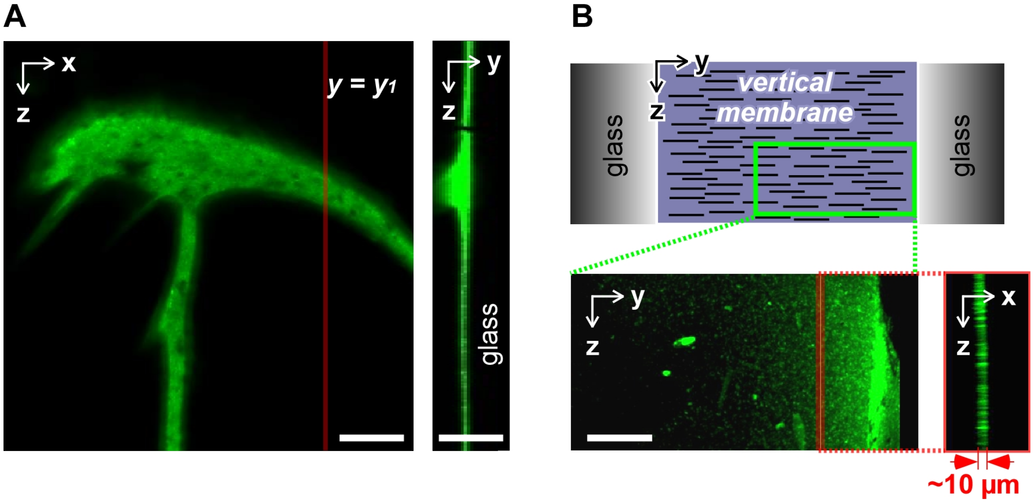


***Figure S5*. Confocal microscopic images of the dried vertical membrane prepared from FITC-conjugated *sacran* solution. A.** Images around the nucleus. **B.** Images around the center of the membrane between the glass slide. All scale bars = 100 $\mu$m.

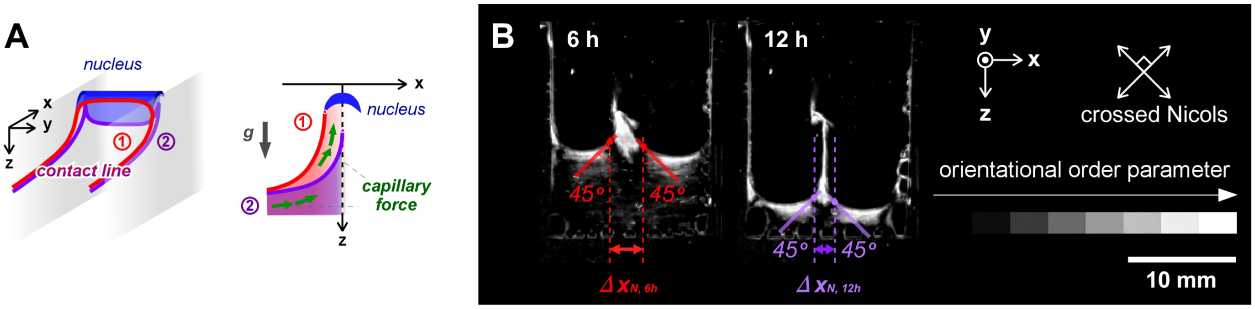


***Figure S6*. Effect of the capillary force in the *Z*-direction near the nucleus. A.** Schematic illustration of the contact line change around the nucleus. **B.** Images around the center of the membrane between the glass slides. Dimensions of the cell: ($\Delta x_0$, $\Delta y_0$, $\Delta z_0$) = (15 mm, 1 mm, ~20 mm). Initial concentration: 0.5 wt%. Drying atmosphere: 60˚C under air pressure.

As shown in **Figure S6B**, the positions in the contact line with 45˚ were marked and the distance between them ($\Delta X_N$) were measured. The validation of the distance ($\Delta X_{N,\,6h}$ = 3.2 mm, $\Delta X_{N,\,12h}$ = 1.9 mm) also supports the mechanism of the **Figure S6A**.

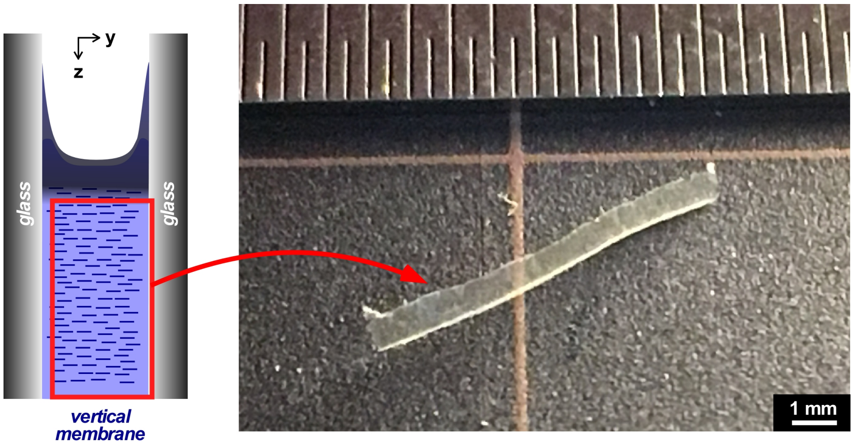


***Figure S7.*** **The dried vertical membrane after pealing off the glass substrates.**

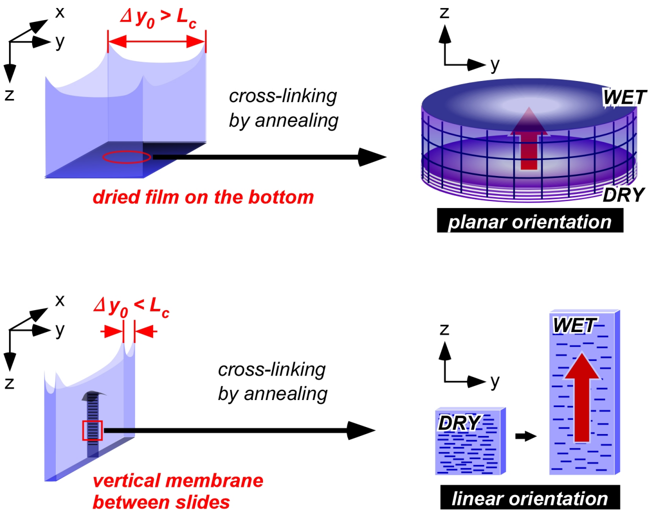


***Figure S8*****. Schematic illustration of drying polymer solution from a cell with top-side-open while controlling the *Y*-thickness, $\Delta y_0$. $L_c$ indicates the critical thickness for vertical membrane formation. A**. Top-side-open cells with a wide *Y*-thickness had a dried film on the bottom, which showed planner orientation. **B**. Top-side-open cell with a narrow *Y*-thickness had a dried vertical membrane between two slides, which showed linear orientation.

The critical thickness, $L_c$, was ~1 mm for 0.5 wt% *sacran* solution. The physical crosslinking point is introduced by annealing the dried membrane at 100°C for 2 h according to previous studies.[2-3]

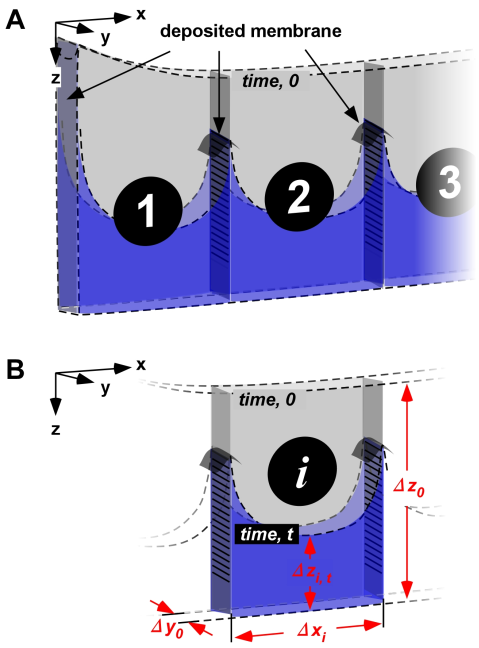


***Figure S9*. Schematic illustration of partitioned spaces by vertical membrane at non-equilibrium state between deposition and hydration during drying from top-side-open cell.** **A.** Space partitioning by multiple nuclei formation. **B.** A partitioned space at time, *t*.

The relation between the polymer concentration in the whole volume and the polymer weight can be described as follows (**Fig. S9A**).

$$C_t \approx \frac{W_0\text{-}\alpha_t}{\Delta x_0 \Delta y_0 \Delta z_t} = \frac{\sum_{i=1}^{n} C_{i,t}}{n} \qquad (1)$$

$$W_0 = \sum_{i=1}^{n} W_i \qquad (2)$$

$C_t$: polymer concentration in a cell at time $t$
$W_0$: initial weight of dissolved polymer in a cell
$W_i$: total weight of dissolved and deposited polymer in a partitioned space ($i$)
$\alpha_{i,t}$: deposition weight of polymer on inside walls and vertical membranes
$\Delta x_0$: $X$-width of the top-side-open cell
$\Delta x_i$: width of a partitioned space ($i$) between membranes
$\Delta y_0$: the $Y$-thickness of the liquid phase
$\Delta z_{i,t}$: height of liquid phase in a partitioned space ($i$) at time $t$
$n$: number of partitioned spaces

The polymer concentration in a partitioned space at time $t$, $C_{i,t}$, can be estimated as follows (**Fig. S9B**).

$$C_{i,t} = \frac{Weight\ of\ dissolved\ polymer\ in\ a\ partitioned\ space}{Volume} \approx \frac{W_i - \alpha_{i,t}}{\Delta x_i \Delta y_0 \Delta z_{i,t}} \qquad (3)$$

## References

[1] M. K. Okajima, Q. T. Nguyen, S. Tateyama, H. Masuyama, T. Tanaka, T. Mitsumata, T. Kaneko, *Biomacromolecules* **2012**, *13*, 4158.
[2] K. Okeyoshi, M. K. Okajima, T. Kaneko, *Biomacromolecules* **2016**, *17*, 2096.
[3] G. Joshi, K. Okeyoshi, M. K. Okajima, T. Kaneko, *Soft Matter* **2016**, *12*, 5515.
